# The Remarkable Dual-Level Diversity of Prokaryotic Flagellins

**DOI:** 10.1128/mSystems.00705-19

**Published:** 2020-02-11

**Authors:** Dalong Hu, Peter R. Reeves

**Affiliations:** a School of Life and Environmental Sciences, University of Sydney, Sydney, New South Wales, Australia; Dartmouth College

**Keywords:** prokaryotic flagellin, evolution and diversity, hypervariable region of flagellin

## Abstract

Flagellin, the agent of prokaryotic flagellar motion, is very widely distributed and is the H antigen of serology. Flagellin molecules have a variable region that confers serotype specificity, encoded by the middle of the gene, and also conserved regions encoded by the two ends of the gene. We collected all available prokaryotic flagellin protein sequences and found the variable region diversity to be at two levels. In each species investigated, there are hypervariable region (HVR) forms without detectable homology in protein sequences between them. There is also considerable variation within HVR forms, indicating that some have been diverging for thousands of years and that interphylum horizontal gene transfers make a major contribution to the evolution of such atypical diversity.

**IMPORTANCE** Bacterial and archaeal flagellins are remarkable in having a shared region with variation in housekeeping proteins and a region with extreme diversity, perhaps greater than for any other protein. Analysis of the 113,285 available full-gene sequences of flagellin genes from published bacterial and archaeal sequences revealed the nature and enormous extent of flagellin diversity. There were 35,898 unique amino acid sequences that were resolved into 187 clusters. Analysis of the Escherichia coli and Salmonella enterica flagellins revealed that the variation occurs at two levels. The first is the division of the variable regions into sequence forms that are so divergent that there is no meaningful alignment even within species, and these corresponded to the E. coli or S. enterica H-antigen groups. The second level is variation within these groups, which is extensive in both species. Shared sequence would allow PCR of the variable regions and thus strain-level analysis of microbiome DNA.

## Abstract (precis)

Bacterial and archaeal flagellins are remarkable in having a shared region with variation in housekeeping proteins and a region with extreme diversity, perhaps greater than for any other protein. Analysis of the 113,285 available full-gene sequences of flagellin genes from published bacterial and archaeal sequences revealed the nature and enormous extent of flagellin diversity. There were 35,898 unique amino acid sequences that were resolved into 187 clusters. Analysis of the Escherichia coli and Salmonella enterica flagellins revealed that the variation occurs at two levels. The first is the division of the variable regions into sequence forms that are so divergent that there is no meaningful alignment even within species, and these corresponded to the E. coli or S. enterica H-antigen groups. The second level is variation within these groups, which is extensive in both species. Shared sequence would allow PCR of the variable regions and thus strain-level analysis of microbiome DNA.

## INTRODUCTION

The bacterial flagellum is a hollow thread-like structure that projects from the surface of many prokaryote cells. The flagellum is made from thousands of flagellin molecules that form a helical supercoil structure, which is attached to a complex membrane-embedded basal body that rotates and imparts this rotation to the flagellum. The rotation causes the flagellar helical supercoil to act as an Archimedes screw to push the cell through its medium or across a surface (1). Three families are recognized based on the distribution of flagella on the cell surface, namely polar flagella confined to one of the poles, peritrichous flagella generally distributed over the surface, and lateral flagella on the sides but not the poles, and the flagella in the three families differ in thickness and also in the pitch of the helix (2). The flagellum is also one of several conserved bacterial components that are recognized by the host innate immune system and is the target of Toll-like receptor 5 (TLR-5), present in both humans and mice, and also of TLR-11 in mice (3). TLR-5 has recently been shown to act by forming a complex with other immune system proteins and flagellin, which then induces an immune response to the bacteria (4). Flagella can also have functions relating to cell surface adherence and, for some pathogens, for infection (5). Flagellins can even have enzymatic activity in their surface-exposed domains (6).

**FIG 1:**
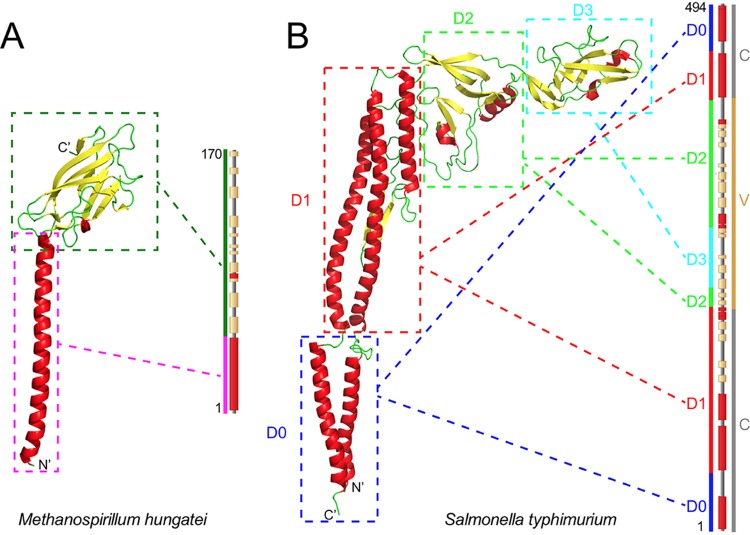
Structure of typcial flagellins. Typical crystal structures of an archaeal flagellin (from Methanospirillum hungatei, PDB accession no. 5TFY) (A) and a bacterial flagellin (from S. enterica serovar Typhimurium, PDB accession no. 3A5X) (B) are shown. In each panel, the flagellin is depicted on the left, with color-coded secondary structures, and boxes with dashed edges link each domain to the corresponding area on the amino acid sequence shown on the right. In panel B, the gene is also shown, with locations of the two conserved regions and the variable region colored gray and dark yellow, respectively.

The flagellum is generated by successive transfer of flagellin molecules into the hollow core of the preassembled basal body and their transport through the basal body and the growing flagellum to the tip of the flagellum, where they extend the flagellum by condensing onto the previous tip flagellin molecule. The flagellin of Salmonella enterica strain LT2 is well documented, and the completed flagellar filament can be treated as a helical assembly of flagellin molecules with roughly 11 molecules per turn or, alternatively, as comprising 11 strands or protofilaments that extend along the flagellum axis (7, 8). There is a single form of flagellin in the S. enterica flagellum, and each molecule has four domains (Fig. 1B) that extend radially from D0 in the center of the flagellum to D3 on the surface (8). Domains D0 and D1 are present in all bacterial flagellins. They are responsible for the structure described above, which assembles into the helical supercoil of the flagellum, and the sequences are conserved within and between species, with divergence similar to that of core genes. Domains D0 and D1 are formed from the ends of the flagellin polypeptide chain, while D2 and D3, which form the most peripheral domain on the surface of the flagellum, are in the center of the polypeptide chain, as shown in Fig. 1B (9). Many species in phyla such as *Proteobacteria* and *Firmicutes* have additional domains which form a highly variable structure on the surface of the flagellum. The only known functions of the D2/D3 domains are as antigens or substrates for phage attachment or interaction with other objects. These probably place limited constraints on variation and also lead to diversifying selection. It is interesting that the transport process has specific requirements for the D0/D1 core domains, but it appears that there are no such constraints on the variable D2/D3 surface domain (10). Kuwajima constructed an E. coli clone lacking the variable region of its flagellin that had no significant effect on filament formation (11).

The diversity of the flagellar surface was first observed as antigenic differences during the development of serology. The Kauffman-White serotyping scheme for S. enterica recognized O and H antigens, of which the H antigen was found to be flagellin (12). The distinction between conserved flanking C1 and C2 regions of the gene (each coding for parts of the D0 and D1 domains) and the variable central V region was then described (13). The variation in the central V region was soon shown to account for H-antigen specificity (14). There are now 114 H antigens in the S. enterica serotyping scheme, but many of them are in sets with closely related sequences (15). Maintenance of this diversity is generally attributed to occasional selection for an alternative structure to avoid host immunity or attack from bacteriophages or predators such as amoeba, for example. In the 1980s and 1990s, Selander and colleagues showed that closely related S. enterica strains could have different H antigens due to recombination, supporting the proposal that natural selection was driving serotype change, as also shown for O antigens (14). It was also shown that while the central regions generally had divergent sequences in antigenically different H types, there was relatively little sequence variation within each H type (14).

Escherichia coli was also used in early studies of flagellar diversity. There are now 53 H antigens recognized in serotyping, most of which were known in the 1970s, when the 50 flagellins of the recognized H antigens were divided into six major morphotypes based on appearance of the flagellum under electron microscopy (16). In 2003, morphotypes were shown to correlate with sequence-based groups of the variable region (9). It was noted that in E. coli, all but one of the flagellin H-antigen-specific variable region sequences are extremely divergent, putting them into H-antigen-specific hypervariable regions (HVRs) (9).

The genes for the flagellar organelle are in several major gene clusters in both E. coli and S. enterica (17, 18), and a shared *fliC* locus has the major flagellin gene in both. The *Salmonella* genus has two species, S. bongori and S. enterica, the latter of which is divided into six subspecies, four of which (subspecies IIIb, II, VI, and I) have an additional FljB flagellin gene at a separate locus. The FliC and FljB flagellins of the biphasic subspecies form a distinct group that is different from the FliC flagellins of the monophasic subspecies (19). Also, some E. coli H-antigen genes map to one of four minor flagellin loci, *flkA* (H3, H35, H36, H47 and H53), *fllA* (H44 and H55), *flmA* (H54) (20), and *flnA* (H17) (21). Strains with these H-antigen genes have inactivated *fliC* genes. It should be noted that these studies used only H-antigen type strains, and to our knowledge, broader studies have not been done. Flagellar synthesis, assembly, and regulation are complex, and E. coli and S. enterica have about 50 flagellar genes in 15 and 17 operons, respectively, with a two-protein master regulator in one operon determining expression of the others in a hierarchic cascade (17, 18). Some species have both polar and peritrichous flagella, and then there are two independent sets of genes and proteins subject to independent regulation (17, 18).

Archaea also have a motility system based on rotation of a spiral-shaped rod. However, the rod and motility system is not homologous to that of flagella, with no shared components. The flagellin equivalent is often called archaellin, but we have retained the use of “flagellin” for both, as they perform the same function. Little is known of archaeal flagellins, but there are two structures (22, 23), and both have an N-terminal α-helix domain equivalent to the bacterial D0 or D1 domain and a C-terminal domain on the surface (22), as shown in Fig. 1A. However, archaeal flagellins are related to type IV pilins (22–25), whereas the bacterial flagellin export system is related to the type III secretion system (26).

Although the diversity of the flagellin variable domain has been described for many species in *Firmicutes* and *Proteobacteria* (27–29), and also in the *Euryarchaeota* (22, 30, 31), the overall picture of prokaryote flagellin diversity is still unclear. And little is known about the origins and evolution of this huge and to our knowledge unique diversity (32). The diversity makes flagellin a promising vaccine target (33) or biomarker (34) and has remarkable potential for high-resolution metagenomics analysis.

Here, we identified 113,285 prokaryotic flagellin sequences from 52 major prokaryote phyla, and the analysis reveals a pattern of diversity that is only easily explained by cross-phylum horizontal gene transfer (HGT) driving the evolution and maintenance of the extreme diversity of bacterial flagellins.

## RESULTS

### Flagellins are extremely diverse and widely distributed in their respective superkingdoms.

**FIG 2:**
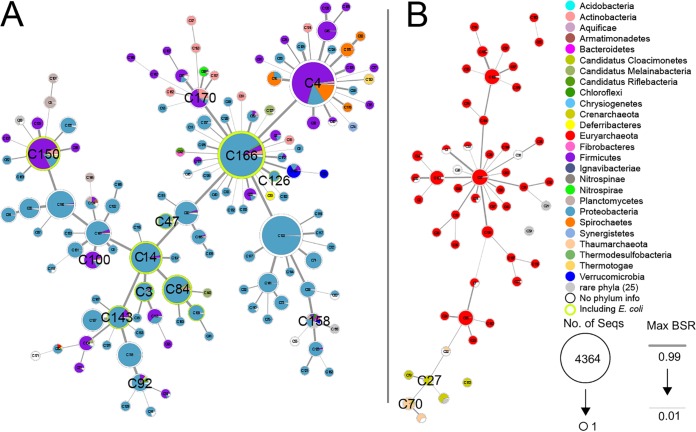
Minimum spanning tree of all flagellin clusters. One bacterial subtree on the left (A) and one archaeal subtree plus three minor groups of archaea on the right (B) are shown by linked pie charts which represent flagellin clusters. In each pie chart, colored segments represent the phyla and their proportions within the cluster, and the circled area reflects the total number of unique sequences in the cluster. The names of clusters mentioned in the text are displayed in a large font. Twenty-five phyla, listed below, have low proportions of observed flagellins, so they are combined and displayed in gray, while the proteins without taxonomic information are displayed in white. The clusters including E. coli flagellins are circled in green. The thickness of branches relates to the distances between clusters (see Materials and Methods). The rare phyla shown in gray are as follows: “*Candidatus* Glassbacteria,” “*Candidatus* Handelsmanbacteria,” “*Candidatus* Margulisbacteria,” *Fusobacteria*, candidate division NC10, *Calditrichaeota*, “*Candidatus* Omnitrophica,” *Coprothermobacterota*, *Elusimicrobia*, *Rhodothermaeota*, “*Candidatus* Latescibacteria,” ”*Candidatus* Magasanikbacteria,” *Chlamydiae*, *Balneolaeota*, *Cyanobacteria*, “*Candidatus* Hydrogenedentes,” “*Candidatus* Raymondbacteria,” “*Candidatus* Kryptonia,” “*Candidatus* Marsarchaeota,” “*Candidatus* Wallbacteria,” “*Candidatus* Lindowbacteria,” “*Candidatus* Rokubacteria,” *Gemmatimonadetes*, *Lentisphaerae*, and *Nanoarchaeota*.

We constructed a database of prokaryotic flagellin protein sequences (see Table S1 in the supplemental material). The database covers 52 phyla (5 archaeal and 47 bacterial), 67 classes, 143 orders, 294 families, and 1,400 genera for the 113,285 proteins in 11,224 species. Flagellins have been identified in almost all major sequenced prokaryotic phyla in the NCBI GenBank database (35). However, while flagella are ubiquitous in many species, some species, such as Acinetobacter baumannii and all *Cyanobacteria*, have different modes of motility and lack flagella. Note that members of the phylum “*Candidatus* Melainabacteria,” phylogenetically related to the *Cyanobacteria*, do have flagella (36). There are 35,898 unique protein sequences (Fig. S1), and it was not possible to use alignment-based approaches for tree generation because of the high number and extreme diversity of the sequences. MMseqs2 (37), which is based on estimated sequence identity, grouped them into 187 clusters (see Materials and Methods), which can be presented as a minimum spanning tree (MSTree) as in Fig. 2. Without alignment of the sequences, we could not separate the roles of the different domains in tree generation. Thus, we looked first at individual species where we have high numbers of flagellin genes and good shared-domain alignments.

10.1128/mSystems.00705-19.1 FIG S1 Modified MStree as shown in Fig. 1 without the information for the three major phyla. To show the distribution of flagellins of rare phyla, the contributions of the three major phyla, *Proteobacteria*, *Firmicutes,* and *Euryarchaeota*, are blanked and moved to the outer circle of each cluster. Twenty-five rare phyla are combined and displayed in gray as described for Fig. 2. Download FIG S1, TIF file, 1 MB. Copyright © 2020 Hu and Reeves. 2020 Hu and Reeves This content is distributed under the terms of the Creative Commons Attribution 4.0 International license.

10.1128/mSystems.00705-19.10 TABLE S1 Proteins used as the flagellin database. Download Table S1, XLSX file, 6.4 MB. Copyright © 2020 Hu and Reeves. 2020 Hu and Reeves This content is distributed under the terms of the Creative Commons Attribution 4.0 International license.

### Details of flagellin diversity in two model species.

The variable D2/D3 regions of the 53 H antigens in E. coli are extremely diverse, with the exception of four pairs. H1 and H12 are very similar, and the other three pairs, H30/H32, H5/H56, and H8/H40, have limited segments of poor alignment in the variable regions. In effect, there are 52 very divergent variable regions (Fig. S2) with few shared segments found in an all-versus-all BLAST search (data not shown). S. enterica has 114 H antigens, which fall into only 26 sets of antigens with HVR-level variation (Fig. S3) that are equally diverse, with none shared with E. coli. However, most exhibit point mutations (single nucleotide polymorphisms [SNPs]) between different H antigens within that set.

10.1128/mSystems.00705-19.2 FIG S2 HVR form-level diversity of E. coli flagellin. The neighbor-joining tree is based on 154 E. coli flagellin protein sequences that are color coded for morphotypes and named by serotype. Multialignment of those sequences ordered by the tree is shown on the right. Download FIG S2, TIF file, 2.4 MB. Copyright © 2020 Hu and Reeves. 2020 Hu and Reeves This content is distributed under the terms of the Creative Commons Attribution 4.0 International license.

10.1128/mSystems.00705-19.3 FIG S3 HVR form-level diversity of S. enterica flagellin. A neighbor-joining tree similar to that in Fig. S2 is shown. Flagellin protein sequences of S. enterica are used to build this tree. The 26 distinct HVR forms are divided by gray lines, and previously defined serotypes and complexes are marked. Download FIG S3, TIF file, 1.8 MB. Copyright © 2020 Hu and Reeves. 2020 Hu and Reeves This content is distributed under the terms of the Creative Commons Attribution 4.0 International license.

This sharp distinction between levels of variation between and within these sets is as clear-cut as the differences between the 90+ chemical elements and between the isotopes of an element. We propose that those in the first category be called hypervariable-region (HVR) forms and the variants within HVR forms be called isoforms. We propose that the HVR forms of E. coli be named, for example, the H1 HVR form, which includes the highly similar H1 and H12 isoforms. Thus, E. coli and S. enterica have 52 and 26 HVR forms, respectively (Fig. S2 and S3), with very different numbers of isoforms identified serologically as H antigens. The probable reason for the higher subdivision levels of the S. enterica HVR forms is that considerable effort was put into finding serological differences between pathogenic strains of S. enterica at a time when serology was the major way to diagnose the presence of pathogenic strains. This involved the use of adsorbed sera to distinguish H antigens of strains with nearly full cross-reactions that were not otherwise distinguished. The typing schemes specify the type strain to be used for each H antigen, and in effect only one antiserum is made for most E. coli HVR forms, but many strains are used for some S. enterica HVR forms. Thus, the sera then need to be adsorbed by other strains to remove antibodies to the shared epitopes. This was not done in E. coli, and the diversity within HVR forms other than the H1 HVR form was revealed only by sequencing. The subdivision of the S. enterica HVR forms was foreshadowed by the complexes described by McQuiston et al. in 2011 (38), and some of these names are used in Fig. S3. This dual-level diversity pattern appears to be widespread, as discussed below.

### Phylogenetics of E. coli flagellin genes.

The 967 unique E. coli flagellin sequences are distributed among nine clusters (see Materials and Methods), marked with light green edges in Fig. 2; in all but one, the *Proteobacteria* predominate. Most HVR forms with morphotypes A, B, and D are in cluster C14, and most of those with morphotypes E, F, and U are in the adjacent C84 cluster. The H37, H48, and H53 flagellins are in adjacent clusters C143, C3, and C47, respectively. The H44 and H45 flagellins are atypical in being in the *Firmicutes*-dominant C100 and C150 clusters, respectively, which are very distant from the two major E. coli flagellin clusters. It is also interesting that E. coli flagellin genes in C166 are only for lateral flagellar flagellins and there are four apparent lateral flagellin genes in the adjacent C126 cluster. However, it should be noted that while the Flag-2 gene cluster (for the lateral flagellin) is present in 20% of E. coli strains, it is usually inactivated (39).

**FIG 3:**
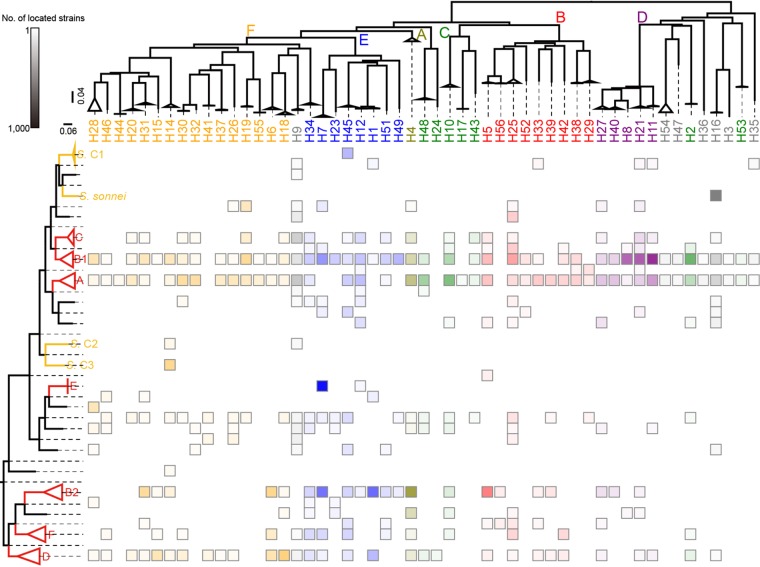
Distribution of E. coli flagellins according to strain phylogenetic groups. The heat map demonstrates the relative numbers of E. coli genomes with each serotype on specific branches. The phylogenetic groups and serotypes are ordered by the phylogenetic trees shown on the left and at the top, respectively (see Materials and Methods). Branches for major E. coli or *Shigella* phylogroups are highlighted in red or yellow, respectively. Morphotypes of each serotype are color coded, and the boxes representing the presence of the serotypes use the same color scheme, with gradation to illustrate the relative numbers of genomes.

Phylogenetically, E. coli strains are divided into major clades named A, B1, B2, C, D, E, and F (40). We also need to include *Shigella* strains, as they are known to be part of the species E. coli, and these were reported to occur in three clades, named C1, C2, and C3, plus three strains not in any of those clades (41, 42). In the 11,162 E. coli and *Shigella* genomes, available in the NCBI RefSeq database (43), there is no relationship between the genome phylogeny and flagellin sequence similarity or morphotype (Fig. 3). An H antigen can be in several clades, which can include strains with quite different modes of pathogenicity. Some clades, such as A and B1, with many sequenced strains, include representatives of nearly all of the H antigens. Clade E, the typical O157:H7 enterohemorrhagic E. coli (EHEC) branch (44), is the exception, with only H7 and H12, but this is probably because the strains sequenced have been selected as being from the well-documented major O157:H7 EHEC lineage, with isolates easily identified as those that ferment d-sorbitol slowly or not at all.

Strains of *Shigella* are nonmotile, and it is interesting that *Shigella* clades always include only one H serotype (there is one exception in the *Shigella* C2 clade). The flagellar gene clusters are generally present in *Shigella* genomes, but flagella are not normally expressed due to mutational deletion or inactivation of the *flhDC* flagella regulatory locus in representatives of all 46 serotypes (45). Loss of flagellin will remove the innate immune response due to TLR-5 or TLR-11, but it is not known how *Shigella* bacteria reach the intestinal wall for invasion without flagella. Inactivation of *flhDC* instead of the *fliC* gene is perhaps because this also avoids synthesis of the flagellar basal body, which would be energetically favorable. The flagellin gene was retained in all 46 strains, and the lack of within-HVR variation is presumed to be due to lack of selection when not expressed.

Detailed species-level diversity of E. coli flagellins is shown by an amino acid multialignment ordered by a neighbor-joining tree based on scores of pairwise alignments of those sequences (Fig. S2). One hundred fifty-four unique E. coli flagellin proteins were selected in this analysis (see Materials and Methods). The extremely low diversity at the two terminals and extremely high diversity in the middle region are clearly shown by the alignment (Fig. S2), and accordingly, the tree has very long inner branches between serotypes but short branches within H antigens, as almost all E. coli H antigens correspond to HVR forms. The contributions of the variable and conserved regions are not visible, but the fit of the tree to morphotypes is probably because of differences in the variable regions, although no similarity between sequences in the same morphotype is shown directly from the multialignment. In Fig. S4 to S6, the three region-based DNA-level maximum-likelihood trees of the 947 unique DNA sequences further support the relationship between sequence and morphotype. Notably, the tree of the variable region shows low branching or evolutionary information between serotypes compared to those of the two conserved regions, but it still groups most serotypes into clusters corresponding to morphotypes, perhaps because of the length variation between morphotypes in this region (Fig. S5).

10.1128/mSystems.00705-19.4 FIG S4 Phylogenetic tree of five prime domains of the E. coli flagellin gene. The maximum likelihood tree of the five prime regions of the E. coli flagellin gene (see Materials and Methods) shows some phylogenetic structures and grouping by morphotypes (indicated by colors). Download FIG S4, TIF file, 1.6 MB. Copyright © 2020 Hu and Reeves. 2020 Hu and Reeves This content is distributed under the terms of the Creative Commons Attribution 4.0 International license.

10.1128/mSystems.00705-19.5 FIG S5 Phylogenetic tree of the hypervariable region of the E. coli flagellin gene. The maximum likelihood tree of the hypervariable region of the E. coli flagellin gene (see Materials and Methods) shows no phylogenetic structure and grouping by morphotypes (indicated by colors). Download FIG S5, TIF file, 2.3 MB. Copyright © 2020 Hu and Reeves. 2020 Hu and Reeves This content is distributed under the terms of the Creative Commons Attribution 4.0 International license.

The E. coli*/Shigella* and S. enterica flagellins resemble those in other *Proteobacteria* and other phyla in having conserved and variable regions and overall diversity, but we are not aware of such studies in other phyla. We selected, for detailed analysis and comparison, the sequences of the phylogenetically distant *Clostridium* group XIVa from the *Firmicutes*, comprising five related species, including Eubacterium rectale, one of the dominant commensal species in the human gut microbiome and also in our *Clostridium* genomes (Fig. S7) (46). The 162 unique proteins are in 37 HVRs, exhibiting a dual-level diversity pattern similar to that seen in E. coli*/Shigella* and *Salmonella*, indicating that this is a widespread pattern in the bacterial superkingdom.

10.1128/mSystems.00705-19.6 FIG S6 Phylogenetic tree of the three prime domains of the E. coli flagellin gene. The maximum likelihood tree of the three prime regions of the E. coli flagellin gene (see Materials and Methods) shows some phylogenetic structures and grouping by morphotypes (indicated by colors). Download FIG S6, TIF file, 2.3 MB. Copyright © 2020 Hu and Reeves. 2020 Hu and Reeves This content is distributed under the terms of the Creative Commons Attribution 4.0 International license.

10.1128/mSystems.00705-19.7 FIG S7 HVR form-level diversity of *Clostridium* group XIVa flagellins, generated as described for Fig. S2 using the 162 unique flagellin protein sequences of *Clostridium* group XIVa species. The sequences from the five species are color coded. Thirty-seven HVR forms are identified and are separated by gray lines. Download FIG S7, TIF file, 1.4 MB. Copyright © 2020 Hu and Reeves. 2020 Hu and Reeves This content is distributed under the terms of the Creative Commons Attribution 4.0 International license.

### Distribution of flagellin clusters.

The 187 clusters defined by MMSeqs2, with 1 to 4,364 unique sequences, are each specific to either the bacterial or archaeal superkingdom. The bit score ratio (BSR) between the most similar two sequences in each pair of linked clusters defines the distance between those clusters. A minimum spanning tree based on the distance links clusters into one bacterial supercluster, one archaeal major supercluster, and three archaeal minor superclusters (Fig. 2).

The 187 clusters in the minimum spanning tree (Fig. 2) are dominated by the top three phyla, the *Proteobacteria*, *Firmicutes*, and *Euryarchaeota*, which have 24,234, 7,500, and 994 unique sequences in 95, 51, and 44 clusters, respectively, while 26 phyla each have fewer than 10 unique sequences (Fig. S8). The top three phyla have over 90% of the sequences, and not surprisingly dominate in all of the larger clusters.

10.1128/mSystems.00705-19.8 FIG S8 Details of flagellin diversity in each phylum. The bar chart shows detailed information of the number of clusters (red), the number of sequenced genomes (green), and the number of unique flagellin proteins (blue) for each phylum shown in Fig. 3. Download FIG S8, TIF file, 2.4 MB. Copyright © 2020 Hu and Reeves. 2020 Hu and Reeves This content is distributed under the terms of the Creative Commons Attribution 4.0 International license.

**FIG 4:**
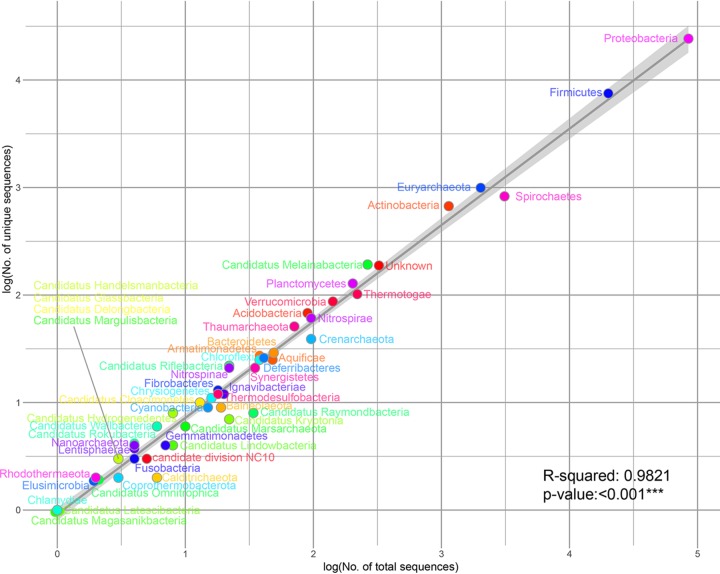
Diversity of flagellins in each phylum. The logarithms of unique flagellin protein numbers and total genome sequence numbers for each phylum are marked by circles on the two-dimension point chart. Each circle is color coded for the phylum. The regression line indicates the average level of diversity within a phylum covering 98.21% of the difference with statistically high significance (*P* value < 0.001). The 95% confidence interval of the regression line is shown as a gray band.

However, the number of unique sequences or clusters does not reflect the real diversity, due to the very low number of sequences in some phyla. We therefore estimated the diversity in each phylum by using the ratio of the numbers of unique and total sequences and found a nearly linear relationship between the two numbers (Fig. 4), which shows that current data are not approaching saturation of unique forms in any phylum. However, the ratio does vary for some phyla, and we will treat the variation as reflecting the underlying diversity. Thus, flagellin diversity may be estimated by comparing the ratio with the regression line in Fig. 4. For example, the *Archaea* contain 1,109 unique sequences from 2,222 observations covering 52 clusters, and the ratio value of 0.5 indicates a higher underlying diversity than that indicated by the 0.31 ratio for bacterial flagellins, which include 34,318 unique sequences from 110,895 observations covering 135 clusters.

In terms of this “unique sequence ratio,” the *Proteobacteria* diversity is estimated to be at the average level, as it is on the line in Fig. 4, whereas the *Firmicutes* and *Euryarchaeota* flagellins are slightly more diverse. Conversely, the next largest phylum, the *Spirochaetes*, has 828 different unique flagellin sequences from 3,108 observations and only five highly related clusters, with the ratio of 0.27 suggesting relatively low diversity (Fig. 4). However, the *Spirochaetes* contain very atypical flagella called “endoflagella” or “axial filaments,” which are not exposed on the cell surface but located in the periplasmic space between the inner and outer membranes and drive a special form of motility (47, 48), and the question arises as to how these are exposed to drive diversification. The *Actinobacteria*, the fourth largest bacterial phylum, form hyphae resembling fungal hyphae that grow by a combination of tip extension and branching of the hyphae; many have a mycelial lifestyle and propagate by forming spores (49). Some of these spores are flagellated and motile (49), and the flagellins are extremely diverse and distributed in many clusters (Table S1). The limited role for the *Actinobacteria* flagella does not eliminate the diversification of its flagellins, and they are on the more diverse side of the line in Fig. 4.

The *Bacteroidetes*, one of the abundant phyla in human and other gut microbiota, are also widely distributed in the environment (50). There is a lack of reports on flagellin diversity of *Bacteroidetes*, and in some metagenomic studies, no motility-related genes were found in this phylum (51, 52). However, according to the estimate in Fig. 4, the *Bacteroidetes* and the *Fibrobacteres* phyla in the same FCB (*Fibrobacteres–Chlorobi–Bacteroidetes*) superphylum (53, 54), have at least average levels of flagellin diversity distributed in 10 and 5 clusters, respectively (Fig. 2 and Table S1). This is a very different situation than in the other very abundant phylum, the *Firmicutes*, in which the diversity has been reported and studied extensively.

## DISCUSSION

There are two very important factors about diversity in the D2 and D3 domains of flagellin, encoded by the V gene regions. First, note that the larger clusters of the bacterial superkingdom all include organisms from highly diverse phyla, 34% (46 of 135) of the bacterial clusters include flagellins from more than one phylum, and eight (C4, C150, C170, C166, C158, C92, C100, and C143) include flagellins from 10 or more phyla (Fig. 2; see Fig. S1 in the supplemental material). Second, there is enormous variable region sequence diversity in all of these clusters, as there are essentially few amino acid sequence similarities between HVR forms, even within clusters. None of the E. coli or *Salmonella* HVR forms has significant sequence similarity with flagellins in any other species. Together, these factors indicate that the extreme diversity of the D2/D3 domains is not due to evolution by mutation during divergence of species or even higher taxa and that there is extensive cross-phylum horizontal gene transfer (HGT) of the flagellin genes, at least over the speciation time frame. However, we do not know the source of any HVR in the two species we studied.

The archaeal clusters are all phylum specific, and clusters, including flagellins from the same phylum, are always linked by branches (Fig. 2 and Fig. S1). Omitting the *Euryarchaeota* sequences in Fig. S1 makes it clear that few phyla are present in more than one cluster, indicating a low HGT rate throughout the superkingdom. For example, the C70 cluster includes flagellins of both *Thaumarchaeota* and *Crenarchaeota* (Fig. S1), but it is linked to C27, which is a *Crenarchaeota*-specific group. Thus, the inclusion of *Crenarchaeota* flagellins in C70 is suggested to be due to a phylogenetic relationship between *Crenarchaeota* and *Thaumarchaeota* (55) rather than an ancient HGT event. This low HGT rate is observed in all archaeal flagellin clusters.

However, the estimation of the archaeal flagellin diversity may be biased due to the limited number of flagellin sequences available. There are only ∼2,000 archaeal flagellins available, compared to over 110,000 bacterial flagellin sequences, and these represent only a few archaeal phyla. In addition, the different origins of bacterial and archaeal flagellins (22–25) means that there is no homology between their flagellins, and the proteins also fold differently. Furthermore, a very high proportion of *Archaea* are not cultivable, and analysis of archaeal flagellins is limited to culturable phyla. Thus, comparison of bacterial and archaeal flagellins may not reflect the real level of their differences or diversity.

### Maintenance of flagellin HVR diversity and turnover of HVR forms.

The maintenance of flagellin HVR form diversity is thought to be based on intermittent positive selection for a replacement H antigen due to factors such as host innate and induced immune reactions that both target this exposed region (56). Other selection factors may include phage that use a flagellin as their receptor for initial attachment. Sequence variation within an HVR is probably in part adaptation that keeps an HVR form competitive. However, the within-HVR form variation seems to never approach the level of that between HVR forms; in other words, intermediate HVR forms are not seen.

There is a high level of synonymous SNPs in the C1 and C2 regions of the 57 E. coli H7 flagellin DNA sequences (Fig. S9), while neutral selection is observed in the hypervariable region. The latter can be attributed to serotype switching by recombination in which the incorporated DNA had one or both ends within C1 and C2.

10.1128/mSystems.00705-19.9 FIG S9 Selective pressure analysis of 57 E. coli H7 flagellin genes. The curves in green, red, and blue represent the cumulative numbers of synonymous mutations, nonsynonymous mutations, and indels crossing the protein sequence from N prime to C prime. The reference sequence is randomly selected from the 57 unique E. coli H7 flagellin proteins. The scales of mutations and indels are shown on the *y* axis on the left and right, respectively. Download FIG S9, TIF file, 0.2 MB. Copyright © 2020 Hu and Reeves. 2020 Hu and Reeves This content is distributed under the terms of the Creative Commons Attribution 4.0 International license.

In theory, without recombination within each form of the hypervariable region, neutral mutations and also slightly deleterious mutations will accumulate in the variable regions of each HVR, in a process known as “Muller’s ratchet” (57). A major role of recombination is proposed to be allowing such trapped slightly deleterious mutations to be lost, but this may not be available for the variable regions, where recombination is possible only within HVR forms. Some examples of chromosomal degeneration have been observed in nonrecombination regions in some eukaryotic nonrecombinant Y chromosomes (58, 59). It has also been described for human populations that the presence of recombination reduces or slows down the accumulation of the neutral or slightly disadvantageous mutations (60), a process that helps to reduce the levels of deleterious variants (61). A possible explanation for the evolutionary pattern of the flagellin variable region is that the high HGT level of flagellin genes allows the more functional variants to spread more widely and frequently than those with deleterious mutations, and thereby they can displace such mutants. This process is a biological example of what is known as the “Matthew effect” (62). The HVR forms appear to be very stable in both E. coli and S. enterica, as the large increases in genome sequences have not revealed new HVR forms as discussed above, indicating saturation in our discovery of new HVR forms in these species. The enormous difference between HVR forms accordingly accounts for the lack of “transition form” flagellins.

The situation is very different for the O antigen, which involves variation in the O-antigen repeat unit, the major surface polysaccharide. There are 184 O-antigen forms in E. coli and 46 in S. enterica, of which 20 are shared (63). The sequence divergence of shared O-antigen gene clusters suggests that they have undergone random genetic drift since divergence from an O-antigen gene cluster in the last common ancestor. There are also several cases of new O-antigen forms arising by recombination between gene clusters or importing a complete gene cluster from another species (64–67).

### Origins of flagellin HVR diversity.

The 76 HVRs found in E. coli and S. enterica are so diverse that we found no shared blocks of sequence in an all-versus-all nucleotide BLAST search. Thus, there is no evidence to support HVR divergence having occurred within the species, and we conclude that the HVRs were acquired from outside. There are no HVRs shared between E. coli and S. enterica, so full turnover of HVRs occurred in the 100 million years that is commonly estimated for their divergence into two genera. We undertook an analysis of the HVRs of a group of related *Clostridia* genera, which gave an overall pattern very similar to that in E. coli and S. enterica, suggesting that this is a common pattern, and the HVR forms are generally species specific. Other evidence discussed above indicates that in all phyla, the number of HVRs already found is very small compared with the total, and it is possible that the HVR pool in other species is indeed the source for E. coli and S. enterica, but no HVR related to any of the source HVRs for E. coli and *Salmonella* has yet been sequenced. Estimates of the number of bacterial species range from over 5 million (68) to one trillion (69), so the pool of HVRs would be massive.

In contrast to E. coli and S. enterica, *Spirochaetes* flagellins have a large number of unique sequences grouped into only 5 highly related clusters (Fig. 4), suggesting relatively low diversity and a low HGT rate, as discussed above. Perhaps the specialized intraperiplasmic location might promote distinctive evolutionary processes in *Spirochaetes* flagellins. Similarly, the rarely observed flagellin-like genes of *Cyanobacteria*, which always lack flagella (70), have a lower level of diversity than those in the closely related phylum “*Candidatus* Melainabacteria,” in which species normally have flagellar motility (36). Another extreme example is in the PVC (*Planctomycetes-Verrucomicrobiae-Chlamydiae*) superphylum, which includes the *Chlamydiae* as discussed above, while the phyla *Planctomycetes*, *Lentisphaerae*, and *Verrucomicrobia*, which are physiologically diverse and found in a variety of environments (71), have significantly higher flagellin diversity. Thus, it is suggested that environment-specific functional diversity contributes more than phylogenetic diversification in flagellin evolution, as some strains, species, or genera with flagellin redundancy, for example, *Caulobacter*, *Vibrio*, and S. enterica, have flagellin switch mechanisms or regulation (72–74). In contrast, the dual-level diversity pattern has not been reported in phyla with low flagellin diversity comprising hyperthermophilic organisms, such as *Aquificae* and *Thermotogae*, although flagellin diversity is higher in the related phylum *Fusobacteria*, which is either pathogenic or commensal in human gut microbiota and plays a role in human colon cancer development (75).

The archaeal flagellins also have high levels of sequence divergence, but in this case, individual HVRs are present in one or few phyla and there is a very low HGT rate. For example, the flagellins of *Crenarchaeota*, which are found mainly in hot springs (55), are in only five adjacent clusters (Fig. 2), and their flagellin diversity is significantly lower than average. However, it is possible that the lack of within-HVR variation in some phyla may be due to differences in ecology that affect sampling. The E. coli and *Salmonella* flagellins with extensive within-HVR variation are from organisms in mammalian intestines and in many cases are pathogenic and subject to strong immune-system selection for antigenic diversity. Many of the fecal sample sources are from disease outbreak patients, and this would provide multiple strains from the various pathogenic or commensal clades known to exist. The hyperthermophilic organisms discussed above are from environmental samples and do not include cases of related strains. One would expect mutations to accumulate in any HVR form and for this variation to be subject to random genetic drift at least and also to selection for change in some surface-exposed domains. We predict that within-HVR variation will be shown to be a general property of flagellins as more data are reported.

### Global model for flagellin HVR diversity.

It is proposed above that the evolution of flagellin HVR diversity within a species is built on the occasional gain of a new HVR by HGT, repeated at a frequency that has led to *Escherichia* and *Salmonella* having no shared HVRs, but not fast enough for us to see intermediate stages, as the flanking shared D2/D3 domains retain no evidence of the HGT crossover boundaries. The ability of intermittent selection for serotype switching to maintain diversity against random genetic drift will be limited, and as new forms come in, others will be lost. This is a remarkable example of HGT, since if we extrapolate to cover the significant proportion of species predicted to be involved, there would be many thousands of HVRs in the system, presumably having diverged by normal evolution but, perhaps uniquely for a chromosomal domain, occasionally transported to another species. We can only assume that the new HVRs came from other species but that none have yet been present in a sequenced genome. Each species thus exhibits extreme HVR diversity by having a set of flagellin HVRs with different origins. Indeed, if the proposal is correct, individual flagellins may have spent extremely long periods of traditional evolution by mutation and selection in multiple species. Several factors may contribute to this pattern. The variable regions are not known to have any significant function other than giving a flagellin a different surface than that of other flagellins, to allow diversifying selection against phage or immune systems or other agents that react with flagellins.

This would make it possible for a variable region to work well in very divergent species. It would also mean fewer constraints on mutational change than for most protein domains. However, the long-term residence of HVRs within a species suggests that it is not easy for a new HVR to get established and compete with them. This is not surprising, as the new HVR has to migrate from one flagellin gene to another with probably a very different pair of flanking D0/D1 domains. There is quite likely a need to have a completely new variable region located precisely between the conserved domains, to ensure that the whole flagellin folds well to resist enzymatic attack. Also, unlike a new O-antigen gene cluster, the new flagellin gene gets no selective advantage until it is part of a functional flagellin regulatory system. It could not replace a resident flagellin gene from a distance, whereas there are examples of an O-antigen gene cluster functionally displacing a resident gene cluster from a plasmid (66). However, once a new HVR is fully integrated into the local conserved regions, it could move between related species by recombination events with donor fragment ends in these conserved regions. We do have some clues to follow up on flagellin evolution. The nine E. coli flagellin genes at the *flkA*, *fllA*, *flmA*, and *flnA* loci are clearly imports, probably from other E. coli strains or related species, that function in the assembly process. Also, the H1/H12, H30/H32, H5/H56, and H8/H40 pairs of E. coli flagellins have some limited sequence similarity. However, further research is required to determine the basis for successful integration of a new HVR form.

In addition, extremely large flagellins with enzymatic activity in D3 regions may have a different model in diversity pattern (76). However, the selective pressure on those flagellins and the influence on the flagellin diversity are still unclear.

### Potential application of flagellin diversity.

The nature of flagellin diversity also has applications in further studies. The 35,898 sequences include genes from 11,224 species in 52 phyla, and with the exception of species known to lack flagella, the most commonly studied taxa are included. This makes flagellins promising marker genes for determining strain diversity in metagenomic studies. There are good strain identification algorithms based on next-generation sequencing, but these are generally confined to the more dominant species in a sample and cannot identify strains with low abundance. The known sequence diversity of the *fliC* flagellins of S. enterica and E. coli is predicted to allow identification of most strains if full-length gene sequences are used, as there are usually SNP differences for a given H antigen in different clones. High-resolution sequencing of flagellin PCR products based on primers in the conserved ends of the gene should give a strain-level resolution profile of metagenomic DNA. Importantly, species-specific PCR would give equal coverage for high- and low-proportion microbiome species, including species like E. coli, which is hard to detect at all by the traditional 16S rRNA method or the shotgun metagenomic sequencing method, since E. coli is usually less than 0.1% of the bacteria in the human gut (77). This capability has been used to identify strains carrying specific flagellin genes by real-time PCR (RT-PCR) (78, 79).

## MATERIALS AND METHODS

### Construction of flagellin database.

First, all proteins with annotations related to flagellin were extracted from the 1 October 2018 version of the NCBI GenBank nr database (35). This was filtered by the two conserved domains (PF00669 and PF00700) in typical bacterial flagellins or the one conserved domain family, PF01917, for archaea. InterProScan v5.31 implemented the scan, using the Pfam v32 database (80), of the first version database to check the existence of the domains. All of the proteins containing both bacterial flagellin domains, or the archaeal domain, were extracted to generate a second version with only “real” flagellin-related proteins. The third version was built based on the second version database as the seed sequences to fish out more flagellin-like sequences from the whole NCBI nr database using a blastp v2.6.0+ (81) search. Another scan of conserved domains by InterProScan, using both Pfam and PANTHER v14 (82) databases, then checked the completeness of the proteins in the third version database. Only proteins containing both of the bacterial flagellin domains, or the archaeal domain, were retained in the fourth version database.

The flagellar hook-related protein FlgL has high similarity to flagellin and always contains one or both of the two conserved domains. Thus, flagellins are often misannotated as FlgL and vice versa (6). The alignment PTHR42792 provided by the PANTHER database includes two subtypes, PTHR42792:sf1 and PTHR42792:sf2, which are used to distinguish FlgL and flagellin. The FlgL in the fourth version database were filtered out by checking the subtype of alignment PTHR42792 based on the last InterProScan run on the PANTHER database. The last step is manual examination and removal of synthetic construct sequences and poor assemblies from low-quality or metagenomic sequences. The final version flagellin database had 35,898 unique protein sequences covering 113,285 recorded proteins for which accession numbers are shown in Table S1 in the supplemental material.

Taxonomic annotation was done with the NCBI Taxonomy database v2018-Oct-1st (83) according to their accession numbers (Table S1). A further manually filtered set named “Unknown” was used for mislabeled taxonomic information, including bacterial flagellins annotated as eukaryote species or nonmotile species such as Acinetobacter baumannii.

A draft clustering by MMseqs2 clust (37) divided the database into 187 groups. An all-to-all comparison was run by blastp to generate a bit score ratio (BSR) (84) matrix for each pair of the 35,898 proteins. The maximum BSR between sequences from each pair of groups is defined as the distance between the two groups. To demonstrate the relationship of the groups, a minimum spanning tree (MSTree) based on the distance matrix was built and displayed by Cytoscape v3.6.1 (85), as shown in Fig. 2. Based on the database, statistical analysis of the number of unique or all sequenced flagellin proteins in each phylum was performed and displayed by R v3.5.1 to show the distribution of prokaryotic flagellin diversity, as in Fig. 2 and Fig. S1.

The two- or three-domain structures and secondary structures of typical archaeal and bacterial flagellin images (Fig. 1) are from accession no. 5TFY and 3A5X downloaded from the PDB database (http://www.rcsb.org) (86) and are rendered by PyMOL v2.2.3 (87).

### Phylogenetic analysis of flagellin in E. coli, S. enterica, and *Clostridium*.

Because of the low proportion of shared sequence in the middle region, a neighbor-joining tree (Fig. S2), rather than a maximum likelihood tree, was generated by MEGA v7.0.20 (88) with 1,000 times bootstrap and default settings, including 154 high-quality E. coli unique flagellin protein sequences extracted from E. coli genomic sequences in the NCBI RefSeq database v2018-Oct-1st (43). The multialignment of those 154 sequences, shown on the right in Fig. S2, was generated by MUSCLE v3.8.31 (89) to illustrate the detailed diversity at the protein level. The alignments and trees shown in Fig. S3 and S7 for flagellins of S. enterica and *Clostridium* used the same build method.

Twenty-nine E. coli and *Shigella* genomic sequences with distinctly different evolutionary backgrounds were selected from the RefSeq database and used to build a phylogenetic tree (Fig. 3, left subpanel), which was built by the maximum likelihood method using RAxML v8.2.11 (90) with 1,000 times bootstrap and the -m GTRGAMMA parameter. The SNPs used to build the tree and recombination events were called by Mauve v2.3.1 (91) and RecDetect v6.0 (92), respectively. Strains E. coli MG1655 and Escherichia fergusonii ATCC 35469 were chosen for the reference sequence and outgroup, respectively. Most of the 11,162 E. coli and *Shigella* genomic sequences in the RefSeq database were then located on the tree by StrainLocater v1.0 (92). The phylogenetic analyses described above were performed using the SaRTree pipeline v1.2 (92) to generate repeatable and reusable output files, which were uploaded onto the FigShare database at https://figshare.com/s/ac165d520410c994f587. Flagellin gene sequences of the ∼11,000 genomes were extracted and identified by a BLAST search using blastx v2.6.0+ with the 154 E. coli flagellin protein sequences described above as the database. The H serotypes of the 11,162 strains were identified as the best hit against a full set in BLAST results. StrainLocater was used to locate the sequences on the appropriate branch of the tree, and the counts of genomes for each serotype on each branch of the phylogenetic tree are shown as boxes in Fig. 3.

All the sequences in the E. coli flagellin gene database were then cut into three regions (the first 570 bp at the 5′ end and the last 300 bp at the 3′ end as two conserved regions and the remaining middle region as the variable region). The multialignments of the three regions were generated by MUSCLE v3.8.31 based on the amino acid sequences. Three phylogenetic trees of them were then built by RAxML v8.2.11 with 1,000 times bootstrap and the -m GTRGAMMA parameter as in Fig. S4 to S6. The HVR forms shown in Fig. S2, S3, and S7 are defined as when there is no alignment with any other HVRs in the hypervariant region.

### Selective pressure analysis.

The codon multialignment program MUSCLE v3.8.31 was used on the translated sequences, the DNA sequences were then recovered for 57 unique E. coli H7 flagellin genes, and SNAP v2.1.1 (http://www.hiv.lanl.gov) (93) was used to calculate the cumulative curves of average synonymous and nonsynonymous mutation rates codon by codon. This was repeated for the other H serotypes, and the statistics and charts were generated by R v3.5.1 as shown in Fig. S9.
